# External validation of semi-automated surveillance algorithms for deep surgical site infections after colorectal surgery in an independent country

**DOI:** 10.1186/s13756-023-01288-y

**Published:** 2023-09-08

**Authors:** Suzanne D. van der Werff, Janneke D.M. Verberk, Christian Buchli, Maaike S.M. van Mourik, Pontus Nauclér

**Affiliations:** 1 Department of Medicine Solna, Division of Infectious Diseases, Karolinska Institutet, Stockholm, 171 77 Sweden; 2 Department of Infectious Diseases, Karolinska University Hospital, Stockholm, Sweden; 3 Department of Medical Microbiology and Infection Prevention, University Medical Centre Utrecht, Utrecht, the Netherlands; 4 Julius Centre for Health Sciences and Primary Care, University Medical Centre Utrecht, Utrecht, the Netherlands; 5 Department of Epidemiology and Surveillance, Centre for Infectious Diseases Control, National Institute for Public Health and the Environment, Bilthoven, the Netherlands; 6 Department of Molecular Medicine and Surgery, Karolinska Institutet, Stockholm, Sweden; 7 Department of Pelvic Cancer, GI Oncology and Colorectal Surgery Unit, Karolinska University Hospital, Stockholm, Sweden

**Keywords:** Automated surveillance, Algorithms, Colorectal surgery, Healthcare-associated infections, Surgical site infections, Validation

## Abstract

**Background:**

Automated surveillance methods that re-use electronic health record data are considered an attractive alternative to traditional manual surveillance. However, surveillance algorithms need to be thoroughly validated before being implemented in a clinical setting. With semi-automated surveillance patients are classified as low or high probability of having developed infection, and only high probability patients subsequently undergo manual record review. The aim of this study was to externally validate two existing semi-automated surveillance algorithms for deep SSI after colorectal surgery, developed on Spanish and Dutch data, in a Swedish setting.

**Methods:**

The algorithms were validated in 225 randomly selected surgeries from Karolinska University Hospital from the period January 1, 2015 until August 31, 2020. Both algorithms were based on (re)admission and discharge data, mortality, reoperations, radiology orders, and antibiotic prescriptions, while one additionally used microbiology cultures. SSI was based on ECDC definitions. Sensitivity, specificity, positive predictive value, negative predictive value, and workload reduction were assessed compared to manual surveillance.

**Results:**

Both algorithms performed well, yet the algorithm not relying on microbiological culture data had highest sensitivity (97.6, 95%CI: 87.4–99.6), which was comparable to previously published results. The latter algorithm aligned best with clinical practice and would lead to 57% records less to review.

**Conclusions:**

The results highlight the importance of thorough validation before implementation in other clinical settings than in which algorithms were originally developed: the algorithm excluding microbiology cultures had highest sensitivity in this new setting and has the potential to support large-scale semi-automated surveillance of SSI after colorectal surgery.

## Background

Healthcare-associated infections (HAIs) pose a major burden on the healthcare system, and result in increased morbidity, mortality, prolonged hospital stay, and additional costs [1–3]. HAIs yearly affect nearly four million patients in acute care hospitals in Europe and surgical site infections (SSIs) account for around 18% of all HAIs, annually affecting more than 500,000 patients [3]. After colorectal surgery, up to or more than 30% of patients develop an SSI [4].

Continuous surveillance with feedback to healthcare personnel and stakeholders is essential to allocate the required resources and assess the effect of interventions to prevent HAIs. Traditional HAI surveillance is often based on time-consuming and resource-intensive manual review of patient records, which is also prone to subjective interpretation and surveillance bias [5–7]. Automated surveillance methods that re-use electronic health record (EHR) data are being developed and considered an attractive alternative to this manual surveillance as it will reduce workload and generates standardised and continuous surveillance results [6, 7]. However, surveillance algorithms need to be thoroughly validated before being implemented in a clinical setting. Their transferability to other countries with different EHR systems and data management than the country of development needs to be assessed before implementation in new settings.

In this study, the aim was to externally validate two existing semi-automated surveillance algorithms for deep SSI after colorectal surgery, developed based on Spanish and Dutch data [8, 9], in a Swedish setting.

## Methods

This retrospective study used EHR data from the Karolinska University Hospital (KUH) stored in the 2SPARE (2020 started Stockholm/Sweden Proactive Adverse Events REsearch) database. KUH is a tertiary care academic center with 1,100 beds divided between two hospitals (Huddinge and Solna), which serves the population of Region Stockholm (2.3 million inhabitants). The study was approved by the Regional Ethical Review Board in Stockholm (no. 2018/1030-31).

**Fig. 1:**
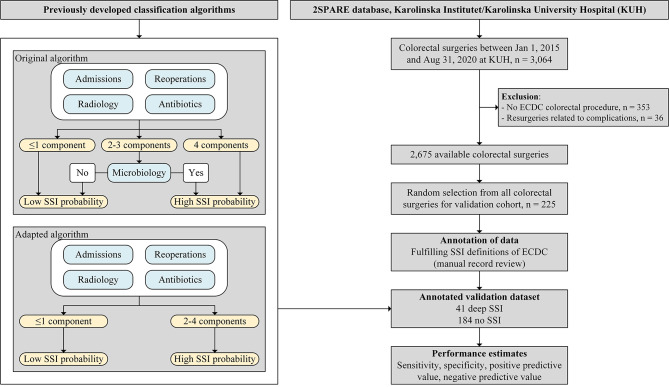
Flow chart of study and flow diagram of classification algorithms for deep surgical site infection. (*SSI: surgical site infection; ECDC: European Centre for Disease Prevention and Control. Admissions: Length of stay ≥ 14 days or 1 readmission to original department or in-hospital mortality within follow-up (FU) time (= 45 days after surgery). Reoperations: Any reoperation by original surgery specialty within FU time. Radiology: ≥1 orders for CT scan within FU time. Antibiotics: ≥3 consecutive days of antibiotics (ATC J01) within FU time, starting after day 1. Microbiology: ≥1 culture taken from relevant body sites within FU time, excluding cultures taken on any day prior to day 1. Original classification algorithm: Figure originally published in van Rooden et al.* [8], *adapted and used with permission. Adapted classification algorithm: Figure originally published in Verberk et al.* [9], *adapted and used with permission*)

With semi-automated surveillance patients are divided in low- and high-probability cases where low-probability cases are automatically regarded as no SSI while high-probability cases undergo manual record review to determine SSI status [6]. Two existing semi-automated classification algorithms to assess deep SSI and/or organ/space SSI, from here on together referred to as deep SSI, were validated (Fig. 1):

1. Original classification algorithm of van Rooden et al., i.e., probability of having a deep SSI based on (re)admission and discharge data, mortality, reoperations, radiology orders, antibiotic prescriptions, and microbiology cultures [8];
2. Adapted classification algorithm according to Verberk et al., i.e., probability of having a deep SSI based on the original classification algorithm without the microbiology component [9].

The algorithms’ performance was assessed in a validation cohort of 225 colorectal surgeries selected via simple random sampling from the 2,675 performed surgeries in the period January 1, 2015 until August 31, 2020 (Fig. 1). Patient and surgery characteristics were recorded. The outcome of interest and gold standard was deep SSI versus no deep SSI (no SSI or only superficial SSI) within 30 days after colorectal surgery as annotated by two experienced infection control practitioners (ICPs) according to the European Centre for Disease Prevention and Control (ECDC) SSI definitions and guidelines [10]. Twenty cases were reviewed in overlap resulting in almost perfect agreement (95%) with a Cohen’s kappa of 0.87 for SSI classification. Both IPCs were blinded for the algorithm results.

Data acquisition, management and analysis were performed using R statistical software (version 3.6.1) and Python (version 3.7), and in accordance with current regulations concerning privacy and ethics. For algorithm performance, the sensitivity, specificity, positive predictive value (PPV) and negative predictive value (NPV) were assessed. The confidence interval (CI) for these estimates were calculated using the asymptotic variance with Wilson score method.

## Results

Within the validation cohort the median age was 66 year (IQR 55–75), 48.9% (n = 110) were female and the median body mass index was 25.6 (IQR 22.3–29.4). Most surgeries were primary (63.6%, n = 143), open procedures (77.3%, n = 174) and related to malignancy (76.9%, n = 173). The surgeries had a median duration of 316 min (IQR 206–427) and in 41.8% (n = 94) of surgeries a stoma was created. In 36.5% (n = 82) of surgeries the patient had an ASA class ≥ 3 and 24.0% (n = 54) of surgeries had a contaminated or dirty-infected wound classification. Within 30-days after surgery 18.2% (n = 41) of patients developed a deep SSI and 2.2% (n = 5) of the patients died.

Both semi-automated algorithms were applied to the validation cohort. Ordered radiology (47.6%, n = 107) and receiving antibiotic therapy for ≥ 3 consecutive days (41.3%, n = 93) were the most common components. In 38.7% (n = 87) of surgeries ≥ 14 days length-of-stay, readmission and/or mortality was present and in 31.1% (n = 70) a microbiological culture was taken. A reoperation (17.8%, n = 40) was the least common component.

Of the 41 patients with a deep SSI, 34 were classified as high probability by the original algorithm (sensitivity 82.9, 95%CI: 68.7–91.5) while 40 were classified as high probability by the adapted algorithm (sensitivity 97.6, 95%CI: 87.4–99.6) (Table 1). The six deep SSI cases only missed by the original algorithm all scored 2–3 components, but were lacking the microbiology component (in other words, no cultures were obtained). The one deep SSI case missed by both algorithms had none of the algorithm components: this deep SSI was manually assessed based on a clinical note describing pus from the rectal stump. The algorithms would lead to workload reduction for manual surveillance of 74% and 57%, respectively.

**Table 1:** Performance classification algorithms for deep surgical site infections after colorectal surgery according to ECDC definitions

	TP	FP	FN	TN		Sensitivity, % (95%CI)	Specificity, % (95%CI)	PPV, % (95%CI)	NPV, % (95%CI)	% workload reduction
Original algorithm^a^	34	25	7	159		82.9 (68.7–91.5)	86.4 (80.7–90.6)	57.6 (44.9–69.4)	95.8 (91.6–97.9)	73.7
Adapted algorithm ^b^	40	56	1	128		97.6 (87.4–99.6)	69.6 (62.6–75.8)	41.7 (32.3–51.7)	99.2 (95.7–99.9)	57.3

*ECDC: European Centre for Disease Prevention and Control; TP: true positive; FP: false positive; FN: false negative; TN: true negative; PPV: positive predictive value; NPV: negative predictive value*. ^*a*^*Original classification algorithm of van Rooden et al.* [8]: *high or low probability of having a deep surgical site infection (SSI) based on (re)admission and discharge dates, mortality, reoperations, radiology orders, antibiotic prescriptions, and microbiology cultures*. ^*b*^*Adapted classification algorithm according to Verberk et al*. [9]: *high or low probability of having a deep SSI based on original algorithm without microbiology cultures component.*

## Discussion

External validation of two existing semi-automated surveillance algorithms after colorectal surgery in EHR data of a Swedish academic hospital center showed that the adapted classification algorithm, indicating high SSI probability based on (re)admission and discharge dates, mortality, reoperations, radiology orders and antibiotic prescriptions, performed best and outperformed the original classification algorithm which also included microbiology culture data. Only one mild case of deep SSI was missed, which would be hard to detect using only structured data as it was assessed merely based on a free-text medical note.

The original algorithm using microbiology culture data was less useful for this specific clinical setting as microbiological culture practices are not standard in suspected deep SSIs after colorectal surgery. However, this might be different in other settings and highlights the importance of thoroughly validation before implementation and pre-emptively investigating if algorithms correspond with clinical practices [9]. It should be emphasised that checking the algorithm periodically against clinical practice after implementation also remains important [6, 7, 9].

Although the original semi-automated classification algorithm was developed based on Spanish and Dutch data, and validated and adapted in the Netherlands, also within Sweden the sensitivity was high and comparable with previous results [8, 9]. These results confirm the potential of large-scale implementation of both, where especially the adapted algorithm, without microbiology data, has demonstrated robustness within Europe.

Strengths of our study were the independent external validation with the extensive availability of EHR data through which the performance of epidemiological surveillance using real-world, real-time data could be mimicked. Limitations were the usage of data from only one center in Sweden, focus of algorithms on only deep SSI and that absence of active post-discharge surveillance of SSI could result in missed SSI.

In conclusion, the results from this study in Sweden, in conjunction with previous studies in the Netherlands and Spain, indicate that a classification algorithm based on (re)admission and discharge dates, mortality, reoperations, radiology orders and antibiotic prescriptions, could be widely implemented for semi-automated surveillance of SSI after colorectal surgery.

## Data Availability

The datasets generated and/or analysed in this study are not publicly available due to ethical limitations with sharing patient information, but are available from the corresponding author on reasonable request.

## Abbreviations

CI: confidence interval
ECDC: European Centre for Disease Prevention and Control
EHR: electronic health record
HAI: healthcare-associated infection
ICP: infection control practitioner
IQR: interquartile range
KUH: Karolinska University Hospital
NPV: negative predictive value
PPV: positive predictive value
SSI: surgical site infection
